# The Protease Degrading Sperm Histones Post-Fertilization in Sea Urchin Eggs Is a Nuclear Cathepsin L That Is Further Required for Embryo Development

**DOI:** 10.1371/journal.pone.0046850

**Published:** 2012-11-05

**Authors:** Violeta Morin, Andrea Sanchez-Rubio, Antoine Aze, Claudio Iribarren, Claire Fayet, Yves Desdevises, Jenaro Garcia-Huidobro, Maria Imschenetzky, Marcia Puchi, Anne-Marie Genevière

**Affiliations:** 1 Department of Biochemistry and Molecular Biology, Universidad de Concepcion, Concepcion, Chile; 2 Unité Mixte de Recherche 7232, Université Pierre et Marie Curie-Paris6, Banyuls-sur-mer, France; 3 Unité Mixte de Recherche 7232, Centre National de la Recherche Scientifique, Banyuls-sur-mer, France; Laboratoire Arago, France

## Abstract

Proteolysis of sperm histones in the sea urchin male pronucleus is the consequence of the activation at fertilization of a maternal cysteine protease. We previously showed that this protein is required for male chromatin remodelling and for cell-cycle progression in the newly formed embryos. This enzyme is present in the nucleus of unfertilized eggs and is rapidly recruited to the male pronucleus after insemination. Interestingly, this cysteine-protease remains co-localized with chromatin during S phase of the first cell cycle, migrates to the mitotic spindle in M-phase and is re-located to the nuclei of daughter cells after cytokinesis. Here we identified the protease encoding cDNA and found a high sequence identity to cathepsin proteases of various organisms. A phylogenetical analysis clearly demonstrates that this sperm histone protease (SpHp) belongs to the cathepsin L sub-type. After an initial phase of ubiquitous expression throughout cleavage stages, SpHp gene transcripts become restricted to endomesodermic territories during the blastula stage. The transcripts are localized in the invaginating endoderm during gastrulation and a gut specific pattern continues through the prism and early pluteus stages. In addition, a concomitant expression of SpHp transcripts is detected in cells of the skeletogenic lineage and in accordance a pharmacological disruption of SpHp activity prevents growth of skeletal rods. These results further document the role of this nuclear cathepsin L during development.

## Introduction

Recent data supports the notion that cathepsin L, and potentially other cysteine proteases, play important but poorly understood roles in regulated nuclear proteolysis. An endogenously produced nuclear serpin inhibitor of cathepsins, MENT (myeloid and erythroid nuclear termination stage-specific protein), has been first reported to induce a strong repression on cell proliferation [1]. Later on, a cathepsin L has been shown to localize in nuclei where it plays a role in the proteolytic processing of the transcription factor CDP/Cux [2]. More recently, cathepsin L has been demonstrated to cleave histone H3 in mouse embryonic stem cells [3]. These nuclear functions of cathepsin L were initially unexpected in mammals as this enzyme was originally described as a lysosomal protease [4].

We previously reported that an inhibition of the activity of a protease of the cathepsin type disturbs DNA replication and prevents mitosis in the early mitotic cell cycles of sea urchin embryos [5]. We subsequently showed that a cathepsin L protease is necessary for mitotic chromosomes decondensation during cleavage cell cycles of these embryos [6]. These suggested that proteases of the cathepsin L type should specifically proteolyze proteins essential for cell division in early embryos.

On the other hand, male chromatin remodelling is required for initiation of the cleavage cell cycles triggered by fertilization. In sea urczhin, this event involves the replacement of sperm histones (SpH) by maternally inherited cleavage stage (CS) histone variants [7]. The SpH are released from male chromatin and subsequently degraded by a nuclear cysteine protease that catalyzes SpH proteolysis and leaves the CS histone variants unaffected [6], [8]. This SpH protease (SpHp) is present as an inactive precursor in the nucleus of unfertilized eggs and was found to be activated and mobilized into male pronucleus after fertilization [5]. It persists in the nucleus of the zygote during the S phase of the initial cell cycle and co-localizes with α-tubuline in the mitotic spindle during mitosis of the first cleavage division. The inhibition, either pharmacologically or with antibodies, of this protease after insemination blocks the SpH degradation that normally follows fertilization, severely disturbs DNA replication and prevents progression toward mitosis aborting the early development at the initial cleavage division [5], [9].

We report here that the protein responsible for SpH proteolysis is a cathepsin L protease. This cathepsin is not only necessary for SpH degradation but it also persists at later embryonic stages with a specific pattern of mRNA expression suggesting a peculiar role during development.

## Materials and Methods

### Animals and handling of gametes

Sea urchins *Sphaerechinus granularis* were collected in the Mediterranean Sea (Banyuls-sur-mer, France) and maintained until use in running sea water. No specific permits were required for the described field studies. Spawning was induced by intracoelomic injection of 0.2 M acetylcholine. Eggs were collected in sea water, filtered through a 100 µm nylon sieve and washed three times with filtered (0.22 µm) sea water (FSW). Eggs were stored at 19°C until use, while sperm was collected and kept concentrated at 4°C. For fertilization, sperm was diluted 10^5^ fold in a 5% (v/v) egg suspension in FSW, conditions which prevented polyspermy. Only batches with at least 95% fertilized eggs were further used. Embryos washed in FSW were maintained under slow agitation in 100 ml volume at 19°C until used. For pharmacological treatments *S. granularis* embryos were cultured in 24 wells plates at a density of 4000 to 8000 eggs/ml. *Tetrapygus niger* sea urchins were collected from the bay of Concepcion, Chile. Unfertilized eggs, sperm, and zygotes were maintained at room temperature in natural sea water under constant aeration. The cell cycle dynamics in both species are similar with the first cleavage occurring at 90 min at 19°C. Hatching is observed at 15 h p.f. in *S. granularis* embryos (at 19°C), mesenchyme blastulae at 24 h, early gastrula at 30 h, prism at 48 h and pluteus larva at 72 h.

### Identification of SpH protease cDNA and expression of the recombinant mature protein in E. coli

To isolate the cDNA encoding the SpHp of *S. granularis* by polymerase chain reaction (PCR), degenerate primers were designed based on the N-terminal amino acid sequence of the mature protease purified from *T. niger* (5′-CCTCCAYTCGACGGTYTCTGGMACCT) and on the N-terminal amino acid sequence of the corresponding entire protein identified in the *Strongylocentrotus purpuratus* genome (5′-ATGTCCAAYCTMACATTCCTYGTCGC). A sequence of 371 pb was isolated from a *S. granularis* cDNA library in pADNS [10]. The 5′- and 3′-ends of the cDNA were identified by PCR using specific primers deduced from the isolated fragment and primers corresponding to the pADNS vector. PCR products were sub-cloned and sequenced.

The nucleotide sequence encoding mature SpHp (aa 114–336) was amplified by PCR and inserted in pET21a(+) (Novagen) at Not I and Xho I restriction sites. The recombinant protein tagged with 6 His in C-terminus was expressed in *E. coli* (strain BL21) and purified with TALON Metal Affinity Resin according to the manufacturer's instructions (Clontech).

### Alignment and phylogenetic analyses

Two sequence datasets were defined: one containing cathepsin sequences spanning main deuterostomian lineages gathered in GenBank (total dataset), the other is a part of this dataset containing only putative L-like cathepsins, bearing ERFNIN motif (L dataset).

Too divergent sequences were removed in order to keep a sufficient number of residues in the alignment: 42 sequences were kept for the total dataset (length range: 219–484 aa), and 22 for the L dataset (length range: 327–359 aa). Sequences were aligned using MAFFT v5 [11], [12], and ambiguously aligned regions were eliminated with GBlocks [13]. Phylogenetic reconstructions were carried out using Bayesian inference (BI) and maximum likelihood (ML). Bayesian analysis was done with MrBayes 3.1.2 [14], with 4 chains of 10^6^ generations, trees sampled every 100 generations, and burnin value set to 20% of the sampled trees. Sequences were analysed with a mixed amino-acid model [14]. Maximum likelihood reconstructions were carried out using PhyML [15], [16] with an evolutionary model selected via Akaike Information Criterion using ProtTest [17], and validated with 100 bootstrap replicates.

### Antibodies production and immunoblotting

Polyclonal antibodies against the produced recombinant His-SpHp were raised in rabbit by standard immunisation protocol (Eurogentec). The serum was kept in aliquots at -80°C.

For immunoblots, 1 µg of the recombinant mature SpHp and 10 µg of nuclear protein extract from *S. granularis* and *T. niger* were analyzed on SDS/PAGE (Laemmli, 1970) and transferred to PVDF membranes (Millipore). Membranes were saturated overnight in Tris-HCl 50 mM pH 7.5, NaCl 150 mM (TBS), 0.1% Tween containing 5% milk and incubated 2 hours at room temperature in the relevant antibody. After washing three times 10 min in TBS-0.1% Tween, membranes were incubated with a secondary antibody (Pierce-goat anti-rabbit-1∶8000) conjugated to peroxydase. The chemiluminescence signal was visualized using ECL^+^ kit (GE Healthcare) and captured with a camera (Vilber Lourmat).

### Detection of SpH protease mRNA expression

The sequence encoding mature SpHp sub-cloned in pBluescript II SK (Promega) was used to generate sense and antisense riboprobes for Northern-blot and *in situ* hybridization. RNA probes were labeled with digoxigenin-UTP by *in vitro* transcription with RNA polymerase (Roche) following the manufacturer's procedure.

Total RNA from *S. granularis* oocytes and embryos were isolated using Trizol (Invitrogen) and, when indicated, traces of DNA were removed using the Ambion DNA-free kit (Applied Biosystems). RNA preparations were checked on 0.8% agarose gels. For Northern-blot, 15 µg of total RNA samples were separated by a 1% agarose gel electrophoresis containing formamide/formaldehyde and transferred to membrane by standard methods [18]. Probe-target hybrids were detected with an alkaline-phosphatase-conjugated antibody against digoxigenin by chemiluminescence reaction (Roche).

Reverse transcription was carried out on 2 µg of total RNA incubated with 2.5 µM oligo dT, 1 mM dNTPs, 20 Units of RNAsin (Promega), and 0.5 U of reverse transcriptase (M-MLV RT Promega or Primescript RT Takara) in 20 µl. Parallel reactions were performed in the absence of reverse transcriptase to confirm the absence of contaminant DNA. SpH 620 nt amplicons were amplified by PCR (Go-Taq) with 5′-TACGTCACTCCCGTTAAGAATC-3′ and 5′-TTAGACAAGAGGATAGCTGGCTGCA-3′ oligonucleotides according to manufacturer conditions (Promega). PCR products were analysed on 2% agarose gel electrophoresis.

*In situ* hybridization was performed as described previously [19].

## Results

### Molecular cloning and sequence analysis of the sea urchin SpH protease cDNA

To characterize the cysteine protease responsible for SpH proteolysis its cDNA was cloned from an available cDNA library for the European sea urchin *S. granularis*, the species in which the SpHp requirement for cell cycle progression was previously shown [5]. A prior purification of the active enzyme from south-pacific sea urchin *T. niger* zygotes gave information on the N-terminal amino acid sequence of the protein [20]. This sequence (TPGNLQIPDTVDWR-) aligned with the *S. purpuratus* sea urchin genome [21] database (http://www.urchingenome.hgsc.bcm.tmc.edu/) identified a single gene encoding a protein harbouring this conserved peptide (78,6% identities, 11/14 conserved amino acids). The corresponding protein clearly belonged to the cathepsin family of proteases, the peptide being located at position of putative mature domain processing site. The corresponding cDNA was isolated from the *S. granularis* cDNA library by polymerase chain reaction (PCR) using degenerate primers as described in Material and Methods.

**Figure 1:**
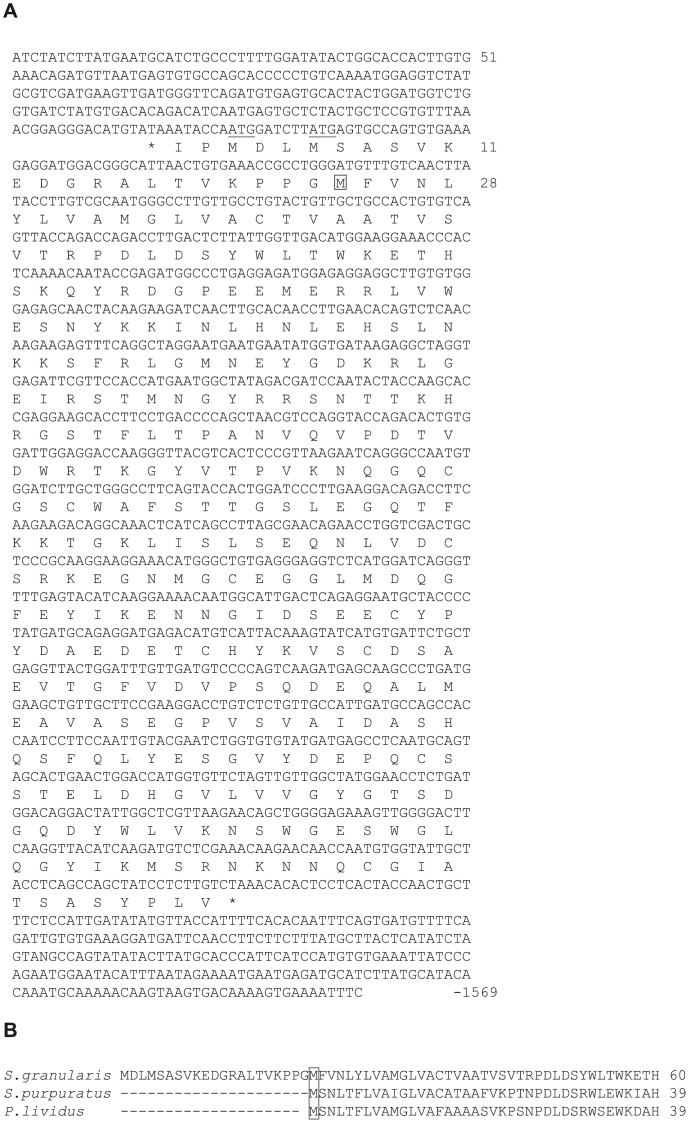
Nucleotide and peptide sequences of *S. granularis* SpH protease . **A**: The sequence of the SpH protease cDNA (1569 nt) is reported with the longest ORF. Two *in frame* ATG codons are found (underlined) before the suggested first Met (M<$>\kern -8.5pt\scale 120%\raster="rg1"<$>) **B**: Alignment of putative N-terminal peptide sequences of *S. granularis, S. purpuratus and P. lividus* SpH-proteases.

**Figure 2:**
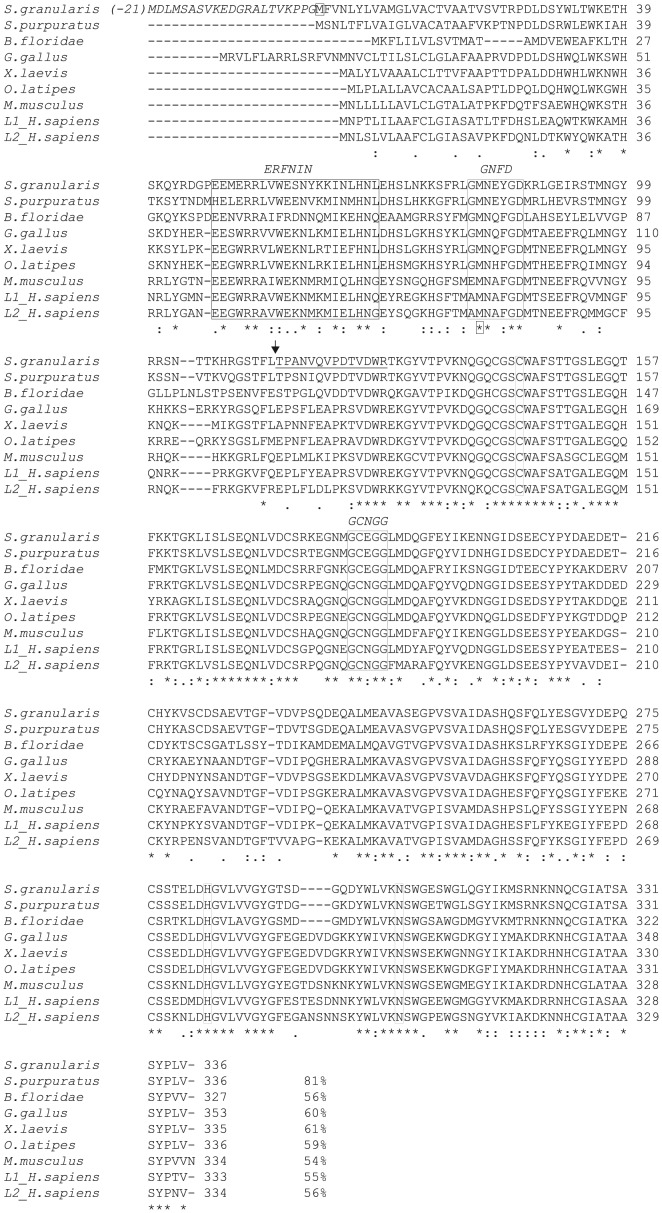
Alignment of SpH proteases sequences (ClustalW). The following sequences are compared: *S. granularis*, *S. purpuratus* (XP_779916.1), cephalochordate *B. floridae* (AAQ01138.1), *G. gallus* (XP_425038.2), *X. laevis* (NP_001087489.1), *O. latipes* (Medaka, gi|50251128|), *M. musculus* (NP_034114.1), *H. sapiens L1* (NP_001903.1) *and L2* (NP_001324.2, also referred as cathepsin V). The 21 encoded amino acids between the first ATG and the putative first Met of the *S. granularis* sequence are indicated in italic. The catalytic triad of Cys-144, His-283 and Asn-303 as well as the ERFNIN, GNFD and GCNGG motives are enframed. The theoretical cleavage site of the prodomain is indicated (<$>\raster="rg2"<$>) upstream from the peptide homologous to the N-terminal sequence of *T. niger* mature SpHp (underlined).

The 1569-bp nucleotide sequence of the cloned cDNA and the derived amino acid sequence of the protein encoded by the longest 1071 bp open reading frame are presented in Fig. 1A. Comparison with orthologue sequences predicted from *S. purpuratus* genome or *Paracentrotus lividus* ESTs (http://goblet.molgen.mpg.de/cgi-bin/webapps/paracentrotus.cgi), Fig. 1B, as well as alignment with cysteine proteases generated by Blast analysis, Fig. 2, suggests that the methionine at position 24 (in Fig. 1A) is the true start of the *S. granularis* protein. The subsequent open reading frame encodes a protein of 336 amino acids with a calculated molecular weight of 37,574. The structure of the SpHp gene suggests that it is translated as a preproenzyme. The open reading frame has a preponderance of hydrophobic residues in the first 14 amino acids which presumably correspond to the leader peptide commonly found at the N terminus of cysteine proteases [22].

The multiple sequence alignment shown in Fig. 2 highlighted the conservation of the catalytic triad of cysteine (Cys-144), histidine (His-283) and asparagine (Asn-303) characteristic for thiol proteinases and motifs characteristics of cathepsin-L like protease family in the putative propeptide region (ERFNIN, GNFD) and the catalytic domain (GCNGG). The theoretical cleavage site of the prodomain between the leucine at position 113 and the threonine at position 114 was predicted from the N-terminal sequence of *T. niger* SpHp. A proline residue found in position 2 of the predicted mature protein has been shown to be conserved in most cathepsin L, two amino acids after the proprotein cleavage site, and may function to prevent N-terminal proteolysis [23]. Of the 14 N-terminal amino acid sequenced from the initially purified *T. niger* cysteine protease, 11 are conserved in *S. granularis* and *S. Purpuratus*, while 89 to 92% identities are found in the catalytical domain of the proteins of the different species (*S. granularis, P. lividus* and *S. purpuratus*) showing the high degree of conservation of this protease.

### Characterization of the cloned SpH protease

**Figure 3:**
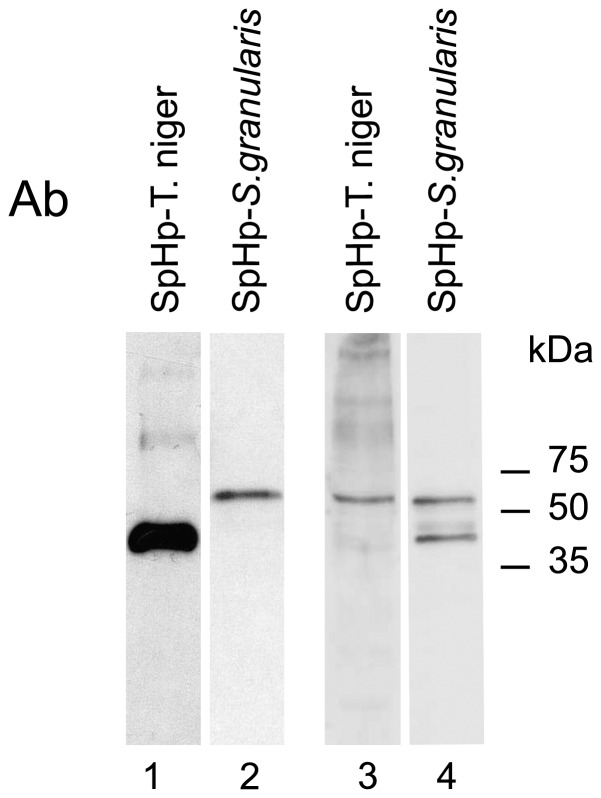
Western blot analysis of native and recombinant SpH proteases. The recombinant mature *S. granularis* SpHp is immunostained by the antibodies raised against the N-terminal peptide of the previously purified *T. niger* SpH protease (lane 1); reciprocally, antibodies raised against the recombinant *S. granularis* mature protease recognized the protein purified by sucrose gradient [8] from *T. niger* fertilized eggs (lane 2). Both antibodies labelled a common electrophoretic band in chromatin extracts from *S. granularis* fertilized eggs (lane 3 and 4). Chromatin in lane 2–4 was prepared from eggs collected 5 min post-fertilization.

The recombinant mature SpHp (from Thr-114 to the C-terminal end) was produced in *E. coli* with a 6-His tag in N-terminus. The protein was purified on Ni column under non-denaturing conditions. We confirmed by western blot (Fig. 3) that the recombinant protein is immunostained by the antibodies directed against the N-terminal peptide sequenced from the purified protease of *T. Niger* [20]. Reciprocally, the antibodies produced in rabbit against the recombinant *S. granularis* protease recognized in western blot the mature cysteine protease purified from *T. niger* fertilized eggs. Moreover, both antibodies labelled a common electrophoretic band in chromatin extracts from *S. granularis* fertilized eggs (Fig. 3). A band of lower molecular weight detected by the new antibodies produced against the full-length mature protein is likely a shorter proteolytic fragment.

From the above data, we concluded that the cloned cDNA encodes the SpH cysteine protease previously purified from sea urchin zygotes and that this protein belongs to the sub-family of cathepsin L-like protease.

### Phylogenetic analysis of echinoderm cathepsin L-like proteins

Based on the overall structure of the proteins, cysteine proteases have been classified in two distinct cathepsin subfamilies, the L-like and the B-like, distinguished by the presence and absence of the ERFNIN motif, respectively [24]. To further investigate the presence of other cathepsin L or L-like proteins in the *S. purpuratus* genome we performed tblastn searches against the GLEAN3 database at HGSC and the sea urchin genome database at NCBI. Three closely related genes were identified which have a lower degree of identity within the encoded N-terminal peptide (14 amino acids) of the putative mature SpHp (respectively 64%-XP 783218, 43%-XP 780653, 29%-XP 780996). They respectively displayed 58 (XP 780653), 56 (XP 783218) and 35 (XP 780996) percent of identity with the putative SpH pre-proprotease. It is to note that in the last predicted gene the Cys 25 which is part of the catalytic triad (C,H,N) conserved in papain [22] is replaced by an alanine. Moreover the conserved Cys22 which forms with Cys63 one of the three disulfide bonds found in the mature papain sequences is not present in this protein. A putative uncharacterized protein (C3YEW8-partial sequence) with similar feature is found in the genome of *Branchiostoma floridae*. Five other predicted gene sequences show high similarities with the previous ones while with some significant differences at the amino acid level. It is currently not possible to conclude if they represent duplicate genes, different alleles of the same genes or genome assembly error, so we chose not to include them in the phylogenetic analysis. The Blast search analyses indicated that the genes mentioned above are the only genes found to contain the highly conserved interspersed ERFNIN motif characteristic of the cathepsin L-like propeptide region in the *S. purpuratus* genome.

**Figure 4:**
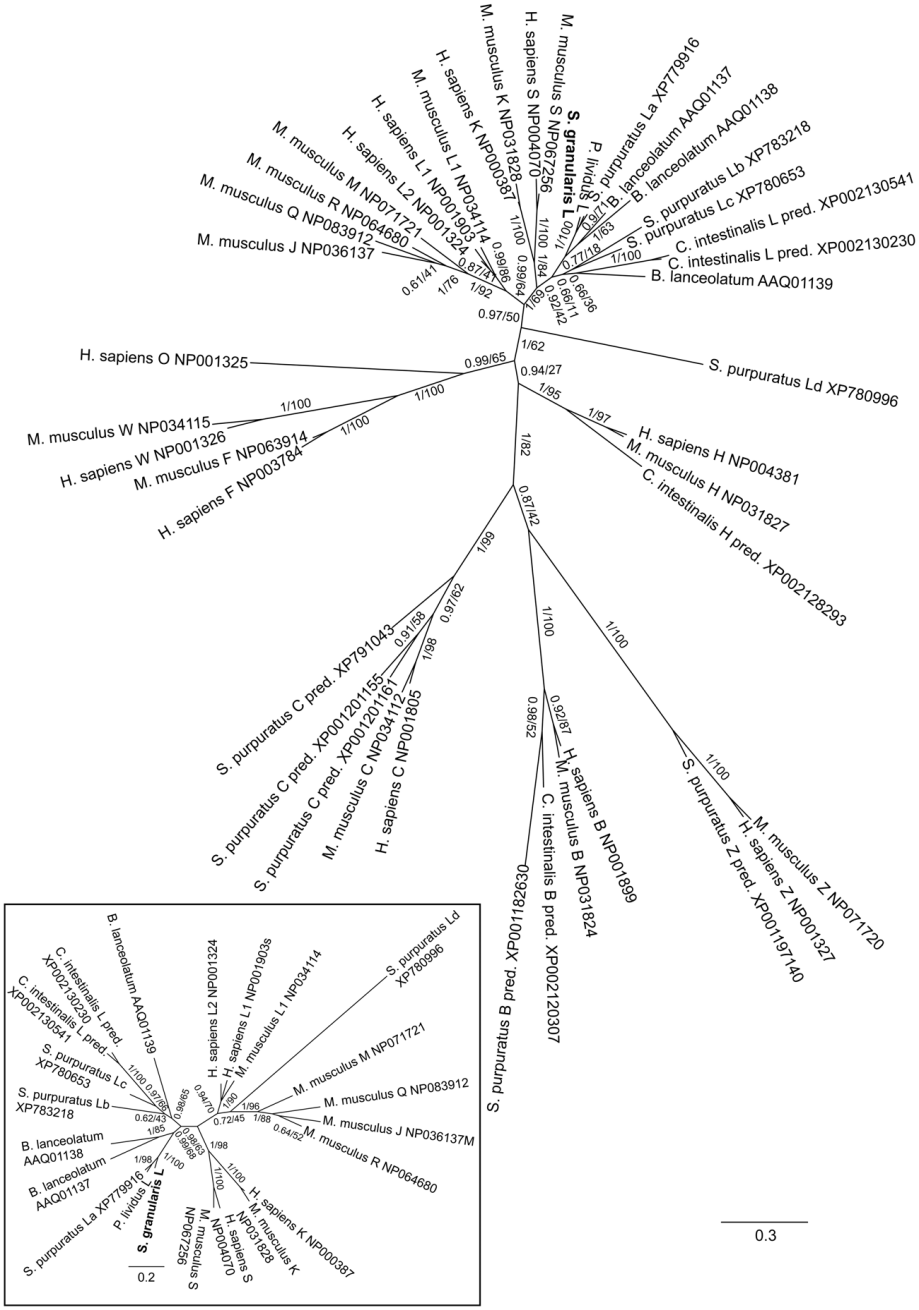
Phylogenetic tree for several deuterostomian taxa. The tree was constructed via Bayesian inference (BI) and maximum likelihood (ML) on cathepsin amino-acid sequences (shown with relevant family, pred.: mentioned as “predicted” in databases, and accession numbers): A: all cathepsin families; B: only L-like cathepsins. The *S. granularis* sequence produced in this study is in bold. Numbers are posterior probability (pp, BI)/bootstrap proportions in % (bp, ML). Internal branches are represented for pp>0.5 or bp>50, scale represents the number of substitutions per site.

Previous phylogenetic analyses performed on large set of cathepsins of the papain family have shown that the B- and L-clades diverged early in the evolution of eukaryotes. A novel phylogenetic analysis was carried out including the newly introduced echinoderm sequences (Fig. 4). Final alignment lengths were 193 aa for the total dataset, and 276 for the L dataset. The phylogenetic trees obtained via BI and ML are fully congruent (Fig. 4). Each cathepsin family clusters in well supported clades. The basal relationships between clades are less clear, but L-like cathepsins are well separated from B-like cathepsin. Cathepsin sequences of L, B, C, and Z type are found in the *S. purpuratus* genome suggesting a conservation of these families across deuterostomians.

### Analysis of expression and function of the SpH protease during sea urchin development

Previous studies have shown that the antibodies elicited against the N-terminal peptide of SpHp purified from *T. niger* labelled cell nuclei of embryos from egg stage to pluteus stage [25]. These results suggest that the enzyme which proteolyzes sperm histones at fertilization may display another yet unknown function during embryonic development. To further investigate the putative role of the newly identified cathepsin L-like gene we further investigated its expression in embryos.

Data from a *S. purpuratus* transcriptome analysis by whole-genome tiling array hybridized with poly-A RNA mixed from egg, early blastula, gastrula and prism stage embryos [21] and expression profiling by whole genome microarrays (http://urchin.nidcr.nih.gov/blast/exp.html) indicate that the presumed SpHp gene is expressed at a somehow constant level from 2 h to 72 h p.f.. In contrast the other putative cathepsin L-like genes are only significantly expressed 72 h p.f., in the pluteus larva.

**Figure 5:**
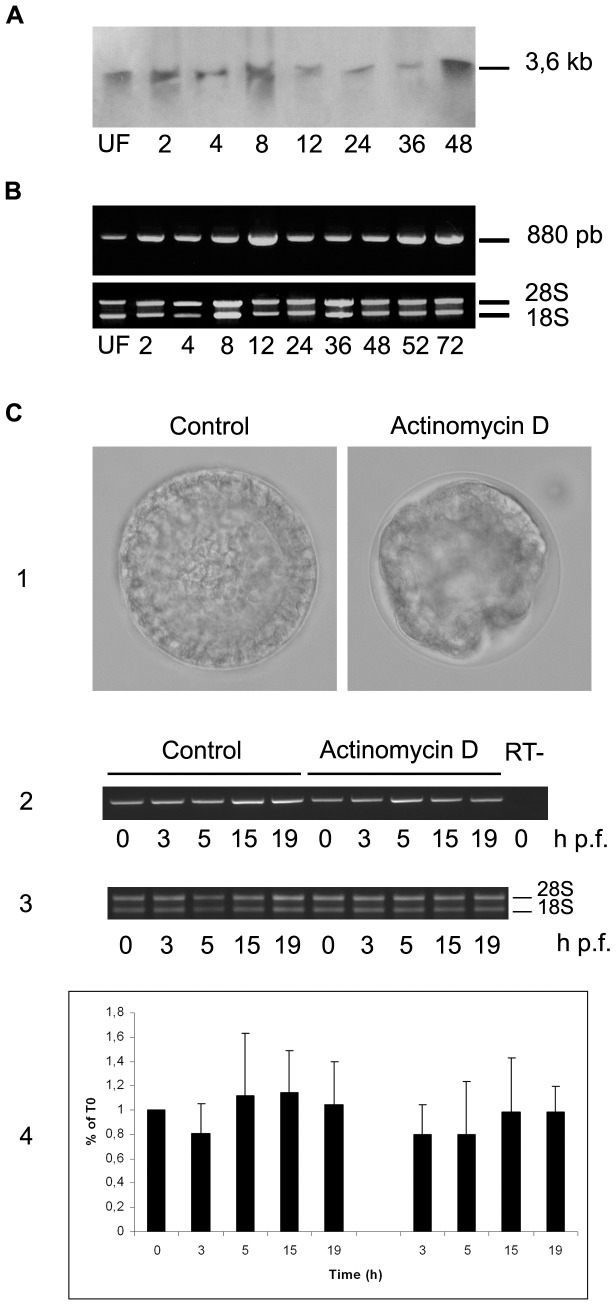
Timecourse of SpH protease mRNA expression along development. Expression of SpH transcripts along development was evaluated by Northern blot analysis (**A**) and semi-quantitative RT-PCR (**B**). PCR was performed as described in Material and Methods using 1 µg of cDNA and 35 amplification cycles (95°C-1 min, 62°C-1 min, 72°C-1 min). The stability of SpH transcripts was estimated (**C**) by treating embryos with actinomycin D (50 µg/ml) 3 min post-fertilization and comparing the abundance of mRNAs in control and treated embryos by semi-quantitative RT-PCR (RT on DNA-free total RNA, PCR with 0.2 µg cDNA, 27 cycles). Inhibition of RNA synthesis with actinomycin D blocks development beyond hatching as visualized in (1) 20 hours p.f.. In these conditions the abundance of SpHp mRNAs remains invariant in treated embryos when compared to controls (1, 2). Quantification of 3 different experiments is reported in (3). Hours post-fertilization are indicated.

The expression level of SpHp transcripts was investigated in *S. granularis* during the first 72 h of development both by northern blot analysis (Fig. 5A) and semi-quantitative RT-PCR (Fig. 5B). A single transcript of 3,6 kb was revealed in northern blot, the abundance of which is rather constant during larva development. The same result was obtained by PCR profiling confirming the constant level of expression of SpHp transcripts in this species too. It is to note that the transcript identified in northern blot is longer than the cloned cDNA, suggesting that 5′- or/and 3′-UTR sequences are not complete. Automated analysis of the *S. purpuratus* genome (NCBI) predicted for example a transcript of 2.7 kb including a 1.6 kb 3′-UTR.

To test the stability of maternal SpHp mRNA, embryos were treated with actinomycin D, an inhibitor of RNA synthesis, and the SpHp mRNA content was evaluated by RT-PCR in control and treated embryos at several timepoints until 20 hours post-treatment (Fig. 5C). While transcription inhibition prevents the embryonic development beyond hatching as expected [26], [27], no significant decay of SpHp mRNA was observed Even if we cannot eliminate a putative residual zygotic transcription in presence of actinomycin D, these results suggest that maternal SpHp transcripts are stable during cleavage stages.

**Figure 6:**
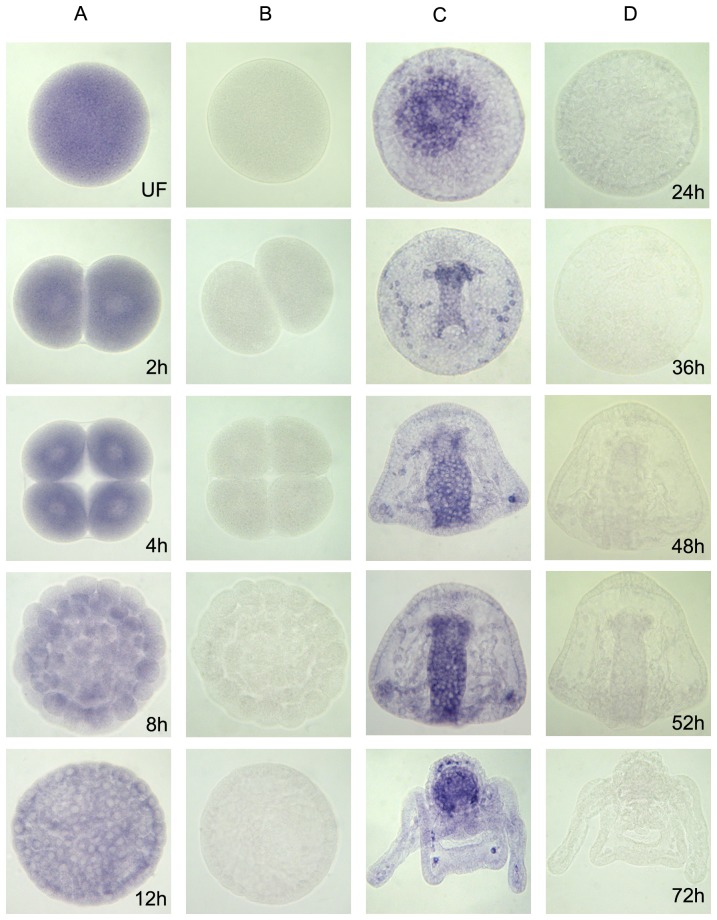
Temporal and spatial expression profile of SpH protease gene during sea urchin embryo development. Results of whole-mount *in situ* hybridization with anti-sense (columns A, C) and sense (columns B, D) probes are displayed for each developmental stage listed at the lower right corner of each panel.

To precisely define the spatial pattern of gene expression, we performed *in situ* hybridization from one-cell stage to pluteus-stage embryos (Fig. 6). SpH mRNAs localized uniformly during cleavage stages and are then enriched at the centre of the vegetal plate of the mesenchyme blastula, in endomesoderm territory. During gastrulation, expression of transcripts becomes localized in the invaginating archenteron. This gut specific pattern continues through the prism and early pluteus stages. In addition, during gastrulation SpHp mRNAs are expressed in the primary mesenchyme cells (PMC), the precursors of skeletogenic cells, while in plutei, a set of discreet cells located in the oral hood within the skeletal rods (subset of mesenchyme skeletogenic cells) also displays a high expression level.

**Figure 7:**
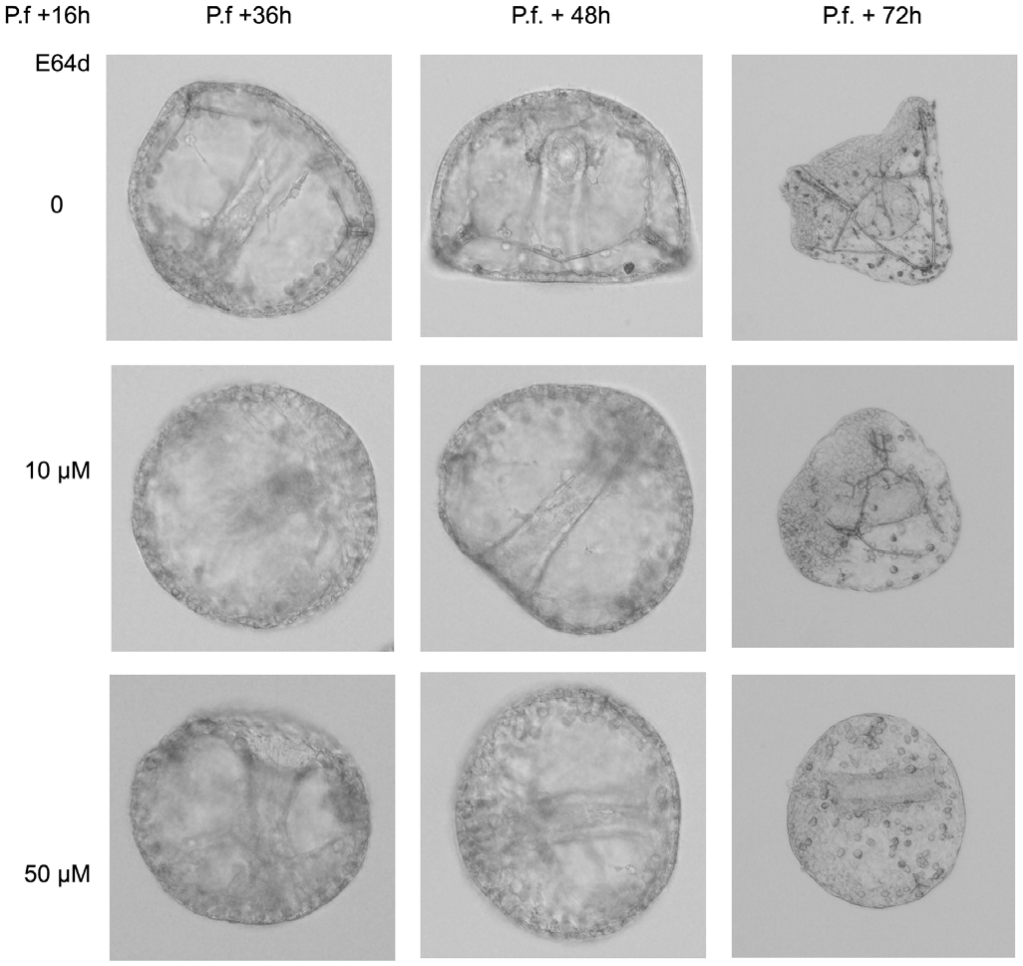
Inhibition of SpHp activity during gastrulation. Sea urchin zygotes were treated after hatching with E64d (10–50 µM) and embryonic development was recorded till 72 h p.f.

To further examine the SpHp function during embryonic development, the enzyme activity was inhibited with the cysteine protease inhibitor E64d (10 and 50 µM) from early swimming blastula stage onward. Till ingression of PMC no morphological differences were observed between treated and control embryos. Afterward, low E64d concentration (10 µM) significantly delays and morphologically disturbs the gastrulation process (Fig. 7). Elongation of the archenteron is affected and skeletogenesis is severely disturbed, a phenotype consistent with the SpHp expression pattern. At higher concentration (50 µM) skeletal rod formation is almost totally prevented.

## Discussion

Proteolytic processing is a key post-translational mechanism that participates in regulation of diverse biological functions including development. While the caspase family of protease is largely involved in the programmed cell death (apoptosis) contributing in shaping the new embryo, the function of other proteases is less documented. Our previous studies have shown that a cysteine protease, SpHp, is responsible for the degradation of sperm histones during the key chromatin remodeling event following sea urchin egg fertilization; its inhibition prevents DNA replication and progression toward mitosis [5], [8]. This proteolytic enzyme remains active in the following cell cycles since immuno-inhibition of SpHp after first mitosis precludes further cell division [9]. N-terminal Edman sequence of the purified protein suggested it belongs to the cathepsin L family. This hypothesis was further supported by a pharmacological approach showing that both antibodies against the N-terminal sequence of the protease and cathepsin L-inhibitor I alter chromatin structure and block cell divisions during early cleavage stages [28]. Here we identified and characterized the gene encoding the SpHp and analysed its expression profile during embryogenesis. These results demonstrate that SpHp is a cathepsin L protease and an essential factor in building of an embryo.

The SpHp identified gene encodes structural features typical of the cathepsin family, including papain family cysteine protease domain and eukaryotic thiol proteases cysteine, asparagine, histidine active sites. Additionally, it exhibits a highly conserved interspersed amino acid motif, ERF/WNIN-like motif, in the pro-peptide region of the protein which is considered as a signature of the cathepsin L proteases [24]. This affiliation to the cathepsin L clade determined from structure and similarity search was confirmed by the phylogenetical analysis.

The maternal SpHp rapidly accumulates in the male pronucleus post fertilization, migrates to mitotic spindle during mitosis, re-localizing to the nuclei of daughter cells in telophase [5], [20]. By western blot using antibodies directed against the catalytic domain of the cloned cathepsin L and *S. granularis* chromatin extracts, we confirmed that this protease is associated to chromatin in newly fertilized eggs. These results provide further illustration of nuclear localization of a cathepsin L endopeptidase and, as far as we are aware, the first example in invertebrates. This evolutionary conserved localization argues in favour of a fundamental role of cathepsin L inside the nucleus.

A few examples of nuclear cathepsin L translocation have been reported either dependent on cell cycle as in NIH3T3 cells [2] or on a pharmacological treatment as in dopaminergic neurons [29]. The mechanism underlying this translocation is poorly understood. Cathepsins destined for the lysosome are synthesized with an N-terminal signal peptide that targets the protein first to the lumen of the endoplasmic reticulum then to the lysosome after a proteolytic activating process [30]. There are several evidences that cathepsin L proteins can be initiated from downstream AUG sites, producing a protein devoided of leader peptide that can be present in the cytosol and nucleus [2], [30]. According to Goulet et al. only cathepsin L isoforms without signal peptides were able to translocate to the nucleus and stimulate proteolysis of the transcription factor CDP/Cux [2]. More recently, using antibodies raised against the sea urchin *T. niger* active SpHp, Puchi et al. [31] uncovered in nuclei of HeLa and Caco-2 cells a 60 kDa SDS-PAGE migrating cathepsin L which degrades histone H1 *in vitro*. In *T. niger* [20] or *S. granularis* fertilized eggs (Fig. 3), these antibodies also labelled a protein with a higher apparent molecular weight than expected for most cathepsin L. It is to note that the recombinant mature SpHp produced either in bacteria (Fig. 3) or in rabbit reticulocyte lysate (data not shown) have slower mobility than the once predicted from the raw amino acid sequence, suggesting a peculiar protein structure or substantial post-translational modifications which could be important for protein function.

While conventional cathepsin L cleaves various proteins efficiently, nuclear cathepsin L exhibits remarkable substrate specificity. Mammalian cathepsin L has been shown to process transcription factor CDP/Cux during the G1/S phase transition, a proteolytic event coupled to cell cycle progression [2]. On the other hand, Histone H3 is proteolytically cleaved by cathepsin L at its N-terminus during mouse embryonic stem cell (ESC) differentiation [3]. This limited nuclear proteolysis process, potentially associated to transcriptional regulation, is controlled by covalent modification of the H3 tail itself and may serve to ESCs to alter epigenetic signatures upon differentiation. In addition, cathepsin L deficiency in knockout mouse fibroblasts was further shown to produce an altered pattern of histone H3 methylation producing a global rearrangement of chromatin [32] and confirming a role of this endoproteinase in regulation of chromatin structure. Replacement of SpH in male pronucleus post-fertilization is also dependent of a proteolysis limited process as SpHp specifically and sequentially degrades sperm histones leaving the maternal cleavage stage histone variants unaffected. This substrate selectivity is also regulated by post-translational modification of the substrates: poly(ADP-ribosylation) of CS histones and phosphorylation of sperm specific histones [33], [34]. During this dramatic sperm chromatin remodelling it has been shown that previous disassembly of nucleosomes is required for complete histone proteolysis however the activity in charge of this displacement remains unidentified [6]. While in that case deposition of maternal histones on sperm DNA is replication independent, it has been suggested that H3 replacement in ESC is S-phase/replication coupled [3]. Both examples of chromatin rearrangement involve specific cathepsin L proteolysis but distinct mechanisms of histones deposition.

Previous pharmacological experiments demonstrated that a cathepsin L inhibitor I-sensitive protease remains necessary beyond the first cell cycle post-fertilization suggesting that this protease activity can play a role in regulation of chromatin structure along embryogenesis [6]. In agreement, northern blot and semi-quantitative PCR analyses demonstrated that the transcripts encoded by the identified cathepsin L gene are constantly expressed during sea urchin development. While the maternal mRNAs are ubiquitously distributed during the cleavage stages, from blastula onward a specific pattern of cell expression is established. The discrete SpHp expression first appears in forming endomesoderm territories like previously observed in zebrafish [35]; this localization persists throughout gastrulation and in early pluteus. In gastrulae the PMC, and later on in plutei a subset of mesenchyme skeletogenic cells, also express SpHp mRNAs. In agreement with this latter localization, pharmacological inhibition of SpHp severely disturbs progression of embryonic development particularly affecting growth of spicules. Thus, after having ensured proteolysis of sperm histones at fertilization contributing to male chromatin remodelling, SpHp continues to play a role during development, disturbing cell cycle during cleavages and controlling more specific cell lineages during gastrulation. Further experiments will be necessary to uncover the proteolytic targets of this enzyme. It can be hypothesize that just at it does with CDP/Cux in mammalian cells, SpHp could cleave some transcription factor required for gastrulation in sea urchin. However, the requirement of this protease all along development rather suggests a control of histones posttranslational modifications resulting in modulation of chromatin dynamics and therefore in regulation of gene expression when zygotic transcription initiates.
